# Unidirectional Mitochondrial Introgression Despite Limited Nuclear Admixture in North American Red‐Backed Voles, *Clethrionomys rutilus* and *C. gapperi*

**DOI:** 10.1002/ece3.72603

**Published:** 2025-11-30

**Authors:** Ben J. Wiens, Jocelyn P. Colella

**Affiliations:** ^1^ Department of Ecology and Evolutionary Biology, Biodiversity Institute University of Kansas Lawrence Kansas USA

**Keywords:** biogeography, hybridization, mitochondrial introgression, mitonuclear discordance, red‐backed voles

## Abstract

Allopatric divergence can result in the evolution of incomplete reproductive barriers, which are put to the test when species come into secondary contact. North American red‐backed voles ( *Clethrionomys rutilus* , *C. gapperi* ) form a broad contact zone, presenting an opportunity to investigate outcomes of secondary contact. Using RADseq data for > 200 red‐backed vole specimens across three transects of the contact zone (Southeast Alaska, British Columbia, Northwest Territories), we test for evidence of admixture and describe the biogeographic history of each region. We find limited evidence for nuclear admixture, but by analyzing nuclear and mitochondrial RAD loci separately, we detect extensive unidirectional mitochondrial introgression. In British Columbia, the pattern of mitonuclear discordance is consistent with a moving hybrid zone, and suggests the possibility of adaptive mitochondrial introgression. In Southeast Alaska, mitochondrial introgression is localized to a smaller geographic region, and involves a distinct *C. rutilus* mitochondrial haplotype that is only found in specimens with *C. gapperi* nuclear genomes. In contrast, we find no evidence for mitochondrial introgression in the Northwest Territories. Together, our results suggest a complex biogeographic history for North American red‐backed voles, mediated by the availability of ice‐free colonization pathways following the LGM and incomplete barriers to reproduction, leading to different outcomes of secondary contact across the continent.

## Graphical abstract

We use RADseq data to investigate the outcomes of secondary contact across three distinct transects of a continental‐scale contact zone between two species of red‐backed voles. We find different outcomes at each transect and document rampant unidirectional mitochondrial introgression.

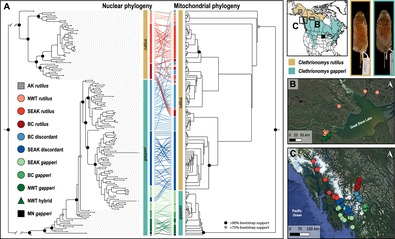

## 1 Introduction

Mitochondrial and nuclear genomes are coevolved to perform key metabolic processes, including aerobic respiration and endogenous heat production (Calvo et al. 2016; Hill 2015). Yet despite the dependence of mitochondria on nuclear‐encoded mitochondrial genes (N‐mt genes) for proper functioning, there are numerous examples of conflicting evolutionary histories between nuclear and mitochondrial genomes, or mitonuclear discordance, across the tree of life (Toews and Brelsford 2012). The preponderance of mitonuclear discordance suggests that species are able to adapt to the disruption of mitonuclear gene networks or that mismatched mitochondrial and nuclear genomes are not always as selectively disadvantageous as may be expected. While the functional consequences of mitonuclear discordance have only been directly tested in a few species, mostly model organisms (Burton et al. 2013; Fishman and Willis 2006; Hanson and Bentolila 2004; Lima et al. 2019; Ma et al. 2016; Meiklejohn et al. 2013; Moran et al. 2024; Pereira et al. 2021), there is a large body of work on the biogeography and population genetics of mitonuclear discordance (Bonnet et al. 2017; Melo‐Ferreira et al. 2009; Sloan et al. 2017; Toews and Brelsford 2012). Describing the biogeography of mitonuclear discordance in natural settings will provide a critical framework for further investigation of how species adapt to mitonuclear discordance in nature.

Mitonuclear discordance can arise through two evolutionary processes. If multiple mitochondrial variants existed in the common ancestor of two species, incomplete lineage sorting (ILS) can lead to conflicting mitochondrial and nuclear evolutionary histories (DeRaad et al. 2023). In this case, mitonuclear incompatibilities are not expected. Alternatively, hybridization can lead to introgression of genetic material across species boundaries (Hibbins and Hahn 2022). Because mitochondrial genomes are inherited matrilineally and without recombination (Harrison 1989), they can be inherited independently from the nuclear genome, potentially leading to mitonuclear discordance. Since mitochondrial and nuclear genomes did not evolve jointly under that scenario, it is possible that mitonuclear incompatibilities may exist (Lima et al. 2019; Moran et al. 2024). While ILS and introgression can both lead to mitonuclear discordance, which process is responsible can often be inferred by considering the spatial and biogeographic context. Mitochondrial introgression is expected to result in a consistent geographic pattern in which individuals with mitonuclear discordance occur at or near the contact zone between the two species (Toews and Brelsford 2012). In contrast, ILS is not expected to lead to a clear geographic pattern because the mitochondrial variants were shared across the common ancestor's range (DeRaad et al. 2023; Toews and Brelsford 2012).

When hybridization occurs following secondary contact, natural selection can lead to differential introgression of nuclear genes and mitochondrial genomes (Bonnet et al. 2017; Gompert et al. 2024; Lindtke and Buerkle 2015; Schield et al. 2017). Indeed, there are numerous examples of hybridization and subsequent mitochondrial introgression across North America (Brelsford et al. 2011; Chavez et al. 2011; Godbout et al. 2012; Graham et al. 2021; Lee‐Yaw et al. 2014; Redenbach and Taylor 2002; Ruegg 2008; Weckstein et al. 2001), many of which can be attributed to recent secondary contact due to the continent's glacial history (G. M. Hewitt 2004). Through the Pleistocene, the climate oscillated between glacial and interglacial periods, with large swaths of North America covered by ice during glacial maxima (Dalton et al. 2020; Raymo et al. 1998). As water became increasingly locked up in glaciers during glacial maxima, sea levels dropped, resulting in the formation of the Bering Land Bridge and other regions of exposed continental shelf along North America's North Pacific Coast (Carrara et al. 2007; Clark and Mix 2002; Hopkins 1973). The last glacial maximum (LGM) occurred approximately 21,000 BP, and during this time, terrestrial fauna persisted primarily north or south of the Laurentide and Cordilleran ice sheets, which combined to extend coast‐to‐coast across much of northern North America (Hewitt 2000). Additionally, some taxa persisted in small ice‐free glacial refugia along the North Pacific Coast and other microrefugia in the high Arctic (Androski et al. 2024; Shafer et al. 2010). Such dynamics through the Pleistocene promoted recurrent bouts of allopatric, refugial divergence followed by secondary contact upon glacial recession.

Northern ( *Clethrionomys rutilus* ) and southern ( *C. gapperi* ) red‐backed voles represent examples of species that evolved in allopatry in response to the onset of Pleistocene glacial cycling ~2 million years ago (Kohli et al. 2014). Beringia, the ice‐free region spanning Alaska, Siberia, and the land bridge that connected them, has been inferred as the primary refugium for *C. rutilus* during glacial maxima (Kohli et al. 2015), while *C. gapperi* is inferred to have persisted south of the continental ice sheets (Runck and Cook 2005). Since the LGM, these species have come into secondary contact across a large region of northern North America spanning Southeast Alaska and Canada. Further, previous work using one mitochondrial gene and one nuclear gene found evidence for mitonuclear discordance in Southeast Alaska (Runck et al. 2009). Here, we leverage museum collections to generate RADseq data for red‐backed vole specimens to quantify mitonuclear discordance and nuclear admixture within this system. We compare results across three transects of the contact zone, located in Southeast Alaska (SEAK), British Columbia (BC), and the Northwest Territories (NWT). We infer fine‐scale biogeographic histories for these species since the LGM and discuss the evolutionary and demographic processes that could be driving differential patterns of mitonuclear discordance across the three transects.

## 2 Methods

### 2.1 Sampling

Red‐backed vole specimens from SEAK, BC, and NWT were holistically collected from 1992 to 2023, archived in, and loaned from one of four museums: University of Kansas Biodiversity Institute (KU), Museum of Southwestern Biology (MSB), University of Alaska Museum of the North (UAM), or University of Wisconsin Zoological Museum (UWZM). Tissue samples for each specimen were collected and preserved on liquid nitrogen in the field before long‐term archival in a museum liquid nitrogen facility. We also included a vole specimen collected outside of the contact zone for each species ( *C. rutilus* from interior Alaska, *C. gapperi* from Minnesota), and two lemming voles ( *Alticola lemminus* ) as an outgroup (Kohli et al. 2014). See Data S1 for a complete list of catalog numbers. All collecting efforts followed ASM guidelines for the use of wild mammals in research (Sikes and The Animal Care and Use Committee of the American Society of Mammalogists 2016) and approved Institutional Animal Care and Use Committee (IACUC) procedures.

### 2.2 DNA Extraction, Library Preparation, and Sequencing

DNA was extracted from cryo‐preserved liver subsamples from 244 specimens following a modified magnetic bead‐based extraction protocol (Rohland and Reich 2012). Library preparation and restriction enzyme‐associated DNA sequencing (RADseq) were performed at the University of Kansas Genome Sequencing Core. Libraries were prepared following a modified RADseq protocol (Andolfatto et al. 2011; Manthey and Moyle 2015). Briefly, DNA samples were digested with the *Nde*I restriction enzyme, given unique barcodes, and size selected for 495–605 base‐pair (bp) fragments. We pooled libraries across four sequencing runs. Each library was sequenced on a single flow cell on the NextSeq2000 Illumina sequencing platform using P2 sequencing chemistry, and generating 100 bp single‐end reads.

### 2.3 Bioinformatic Processing

Raw reads were demultiplexed using the *process_radtags* module in *Stacks* v2.41 (Rochette et al. 2019) and aligned to the *Clethrionomys glareolus* (GCF_902806735.1) reference genome with *bwa* v0.7.17 (Li and Durbin 2009). Alignments were processed in *Stacks* to discover RAD loci (*gstacks*) and call SNPs (*populations*). We included only one SNP per locus (*‐‐write‐single‐snp*) and removed mitochondrial SNPs using *vcftools* v0.1.16. Moving forward, we analyzed nuclear and mitochondrial SNPs separately. We filtered nuclear SNPs for quality and completeness using *SNPfiltR* (DeRaad 2022). We kept SNPs with read depth between 4 and 400, removed SNPs with genotype quality < 30, removed individuals with > 98.40% missing data, and removed SNPs with > 20% missing data. These filters provided a good balance between maximizing the individuals retained while minimizing missing data, and resulted in 7670 nuclear SNPs across 227 individuals with 11.95% overall missing data. We assessed the contribution of missing data to genetic clustering results before continuing with other analyses (Tables S1–S2; Figures S5–S11).

To create an initial dataset of mitochondrial SNPs, we filtered the raw alignments for reads aligned to the *C. glareolus* mitogenome with *samtools* v1.14 (Li et al. 2009). We used *Stacks* to discover SNPs, keeping all SNPs per locus. We used *SNPfiltR* to remove SNPs with read depth < 4 and genotype quality < 30. We recoded heterozygous genotypes as missing data because mitogenomes are haploid. We then removed SNPs with > 90% missing data and individuals with > 90% missing data, resulting in 82 SNPs across 12 RAD loci. We used this dataset to classify mitochondria as either *C. rutilus* or *C. gapperi* by calculating pairwise values of Nei's genetic distance (Pembleton et al. 2013). We identified 202 individuals with *C. rutilus* mitochondria and 28 individuals with *C. gapperi* mitochondria.

To maximize the number of high‐quality mitochondrial SNPs, and because most individuals had *C. rutilus* mtDNA, we used *bwa* to realign all individuals that passed initial mitochondrial quality filters to a *C. rutilus* mitogenome (MK482363.1). We also included the two *A. lemminus* samples as outgroups. We called SNPs in *Stacks* and kept all SNPs per locus. We filtered out SNPs with read depth < 4, genotype quality > 30, and converted heterozygous genotypes (0.37% of all genotypes) to missing data. Within each species separately, we filtered out sites that had > 70% missing data. We implemented an additional filter for *C. rutilus* , removing individuals with > 20% missing data, retaining 112 individuals. We did not impose a similar filter for *C. gapperi* because of the small sample size, leading us to prioritize keeping individuals over more aggressive data filtering. These filters yielded 45 mitochondrial SNPs across 13 RAD loci for 142 individuals (112 *rutilus*, 28, *gapperi*, 2 outgroup) with 14.48% overall missing data. The mitochondrial RAD loci contained portions of the ND1, COX1, ND4, and ND5 genes.

### 2.4 Phylogenetic Analyses

We built a maximum likelihood mitochondrial phylogeny using IQ‐TREE v2.2.2.3 (Minh et al. 2020). Previous work has found the General Time Reversible (GTR) model of nucleotide substitution to perform best for a mitochondrial gene in *Clethrionomys* and that *Alticola* forms a clear outgroup (Kohli et al. 2014). Therefore, we also used the GTR model, corrected for ascertainment bias, specified *Alticola* as the outgroup, and performed 10,000 ultrafast bootstraps (Hoang et al. 2018). To aid interpretation, we made branch lengths proportional to the number of tips under the nodes using FigTree v1.4.4 (Rambaut and Drummond 2012). We also built a maximum likelihood nuclear phylogeny using IQ‐TREE. As input, we used concatenated RAD loci corresponding to the filtered dataset of 7670 nuclear SNPs, resulting in an alignment of 744,813 sites, of which 42,223 were variant. We implemented model testing within IQ‐TREE to select the best model of sequence evolution (Kalyaanamoorthy et al. 2017), performed 1000 ultrafast bootstraps, and rooted the tree at the midpoint. To facilitate comparison to the mitochondrial phylogeny, we built an additional nuclear phylogeny using the same methods, but excluding individuals not represented on the mitochondrial tree. A tanglegram of the nuclear and mitochondrial phylogenies was constructed in SplitsTree6 (Huson and Bryant 2024).

### 2.5 Admixture Analyses

We estimated nuclear ancestry proportions for each individual using *STRUCTURE* v2.3.4 (Pritchard et al. 2000). We removed the outgroup ( *Alticola lemminus* ) from the dataset before running *STRUCTURE*, retaining 6408 SNPs that were still variant across *Clethrionomys*. Our phylogenetic analysis confirmed that *C. rutilus* and *C. gapperi* represent two species, with possible admixture, so we ran the *STRUCTURE* algorithm for 10 replicates of *K* = 2 for 1,000,000 MCMC iterations with a 100,000 iteration burn‐in, where *K* is the number of assumed clusters. To increase computational efficiency, we parallelized the runs using *structure_threader* (Pina‐Martins et al. 2017).

We also tested for admixture by building triangle plots with the R package *triangulaR* (Wiens et al. 2025). We assigned the four northernmost and four southernmost individuals sampled in BC as *C. rutilus* and *C. gapperi* parental populations, respectively. We then created a dataset of ancestry‐informative markers (AIMs) by identifying nuclear SNPs with fixed differences between the parental populations. This yielded 1622 AIMs, which we used to calculate the hybrid index and interclass heterozygosity for every individual.

Initial phylogenetic and population genetic analyses revealed a pattern of mitonuclear discordance in BC (widespread) and SEAK (along the Stikine River), with little to no apparent nuclear introgression. To directly test for nuclear introgression in individuals with mismatched nuclear and mitochondrial genomes, we employed ABBA‐BABA tests to calculate *D*, as implemented in Dsuite v0.5 (Malinsky et al. 2021). For BC, we calculated *D* for each population with evidence for mitonuclear discordance. Individuals were grouped into a population if they occurred within 150 m of each other, resulting in 14 discordant populations, each of which was at least 600 m away from the next. For each discordant population in BC, we used the *C. rutilus* and *C. gapperi* parental populations (as defined above) as P3 and P1, respectively, *A. lemminus* as the outgroup, and the discordant population as P2. With this setup, a positive value of *D* indicates excessive nuclear allele sharing between parental *C. rutilus* and the discordant population. We also tested for nuclear introgression along the Stikine River in SEAK, keeping the same outgroup and P3 as before, but using all individuals along the Stikine River as P2 and all other SEAK individuals with matching *C. gapperi* nuclear and mitochondrial genomes as P1.

### 2.6 Intraspecific Genetic Connectivity

To assess population structure within each species, we refiltered the nuclear SNPs for each species separately. We did this to include RAD loci that were only present in one species, which may not have passed the completeness threshold imposed for the full nuclear SNP dataset. We assigned each individual as *C. rutilus* or *C. gapperi* based on which group comprised at least 80% of its nuclear ancestry, as determined by the previous *STRUCTURE* analysis. One individual (FN414) did not have > 80% ancestry from either species and was therefore excluded from species‐specific analyses. For each species, we implemented the same read depth and genotype quality filters as before, keeping only individuals that passed the previous completeness threshold. We then filtered nuclear SNPs for 80% completeness within each species separately, retaining 3741 SNPs for 94 *C. rutilus* individuals and 7364 SNPs for 130 *C. gapperi* individuals.

To investigate intraspecific genetic relationships, we used the species‐specific nuclear SNPs to run *STRUCTURE* for each species separately. The results of our phylogenetic analysis indicated that intraspecific relationships closely mirrored the three sampled geographic regions (SEAK, BC, and NWT). Therefore, for each species, we ran *STRUCTURE* for 10 replicates of *K* = 3 for 1,000,000 MCMC iterations and a 100,000 iteration burn‐in. We also assessed intraspecific genetic connectivity by estimating effective migration surfaces with EEMS (Petkova et al. 2016). We excluded NWT voles and outgroups from this analysis, as our focus was on addressing connectivity across the topographically complex region of SEAK and BC. For each species, we ran EEMS for 10,000,000 MCMC iterations with a 1,000,000 burn‐in and thinning every 999 iterations, and visually verified convergence at the end of the run. We performed runs with 100, 200, and 400 demes, and found only minor differences in the resulting effective migration surfaces. Using 100 demes provided the highest deme occupancy, so we present those results for both species.

## 3 Results

### 3.1 Phylogenetic Analyses

**FIGURE 1:**
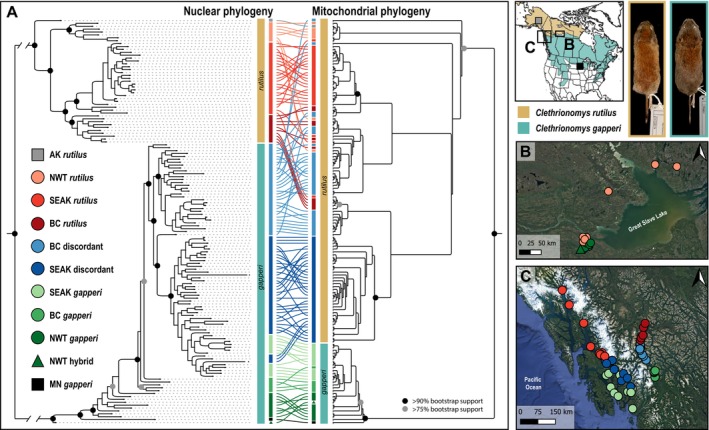
(A) Tanglegram constructed from maximum likelihood phylogenetic trees built for nuclear (left) and mitochondrial (right) RAD loci. Major nodes with > 90% bootstrap support are indicated by black dots and major nodes with > 75% bootstrap support are indicated with gray dots. For the mitochondrial phylogeny, branch lengths are proportional to the number of tips under the node. Only individuals with enough data in both datasets (nuclear and mitochondrial) were used to construct the trees. The colored lines connect individuals across the two trees. Colors and shapes represent geographic regions and the combination of nuclear and mitochondrial species ancestry. For each species, we included a sample from outside of the contact zones, indicated by the gray and black squares. The geographic location of the individuals included in the tanglegram is shown in (B) the map of the Northwest Territories (NWT) sampling region and (C) the map of Southeast Alaska (SEAK) and British Columbia (BC). Representative museum study skins of each species are shown in the top right.

After filtering, 142 individuals were present in both the nuclear and mitochondrial datasets. Using those individuals, we built a tanglegram of nuclear and mitochondrial maximum likelihood trees, which revealed extensive unidirectional mitonuclear discordance (Figure 1). Specifically, 37 individuals from SEAK and 32 individuals from BC had *C. gapperi* nuclear genomes paired with *C. rutilus* mitochondrial genomes, while none had the reverse combination. Discordant individuals from SEAK corresponded to a distinct, well‐supported mitochondrial clade of *C. rutilus* ancestry. Interestingly, that *C. rutilus* mitochondrial clade is only found in SEAK individuals with *C. gapperi* nuclear ancestry. Meanwhile, *C. rutilus* mitochondrial genotypes in BC are shared across individuals regardless of nuclear species ancestry.

For more detailed investigation of nuclear phylogenetic relationships, we retained 227 individuals regardless of whether they had mitochondrial data available. The nuclear maximum likelihood phylogenetic tree recovered a deep split between *C. rutilus* and *C. gapperi* (Figure S1).

**FIGURE 2:**
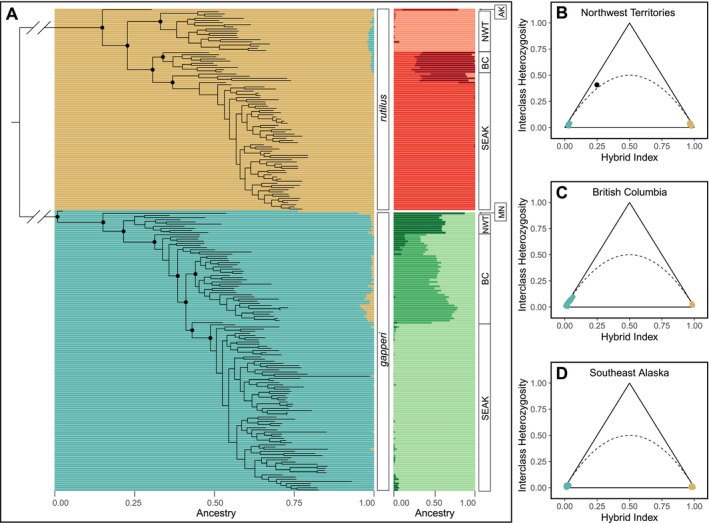
(A) Maximum likelihood phylogenetic tree built using all individuals in the nuclear dataset. Major nodes with > 95% bootstrap support are indicated with a black dot. Species ancestry proportions inferred with STRUCTURE (*K* = 2) are shown horizontally underneath each tip of the tree, such that each row corresponds to the same individual at that tip of the tree. Intraspecific ancestry proportions (*K* = 3 for each species) are shown to the right of the tree, again lining up with the individual at the corresponding tip of the tree. The geographic location for each individual is labeled to the right of the intraspecific ancestry proportions (AK = *C. rutilus* from interior Alaska; MN = *C. gapperi* from Minnesota). Triangle plots are shown for each geographic region separately: (B) Northwest Territories (NWT); (C) British Columbia (BC); (D) Southeast Alaska (SEAK). Teal points indicate individuals with majority *C. gapperi* ancestry and gold points indicate individuals with majority *C. rutilus* ancestry. Only one individual had a hybrid index > 0.1 or < 0.9. That individual, from Northwest Territories, is indicated with a black dot on the triangle plot.

Within *C. rutilus* , the NWT clade diverged first, followed by BC and SEAK (Figure 2A). While each geographic clade is well supported and monophyletic within *C. rutilus* , divergence between BC and SEAK is very shallow. Within *C. gapperi,* the NWT clade was also first to diverge and forms a well‐supported clade, followed by shallow, stepwise divergence across BC and SEAK. There is one individual from NWT with a high proportion of mixed nuclear ancestry, which occurs on a short branch sister to the rest of the *C. gapperi* clade.

### 3.2 Admixture Analyses

**FIGURE 3:**
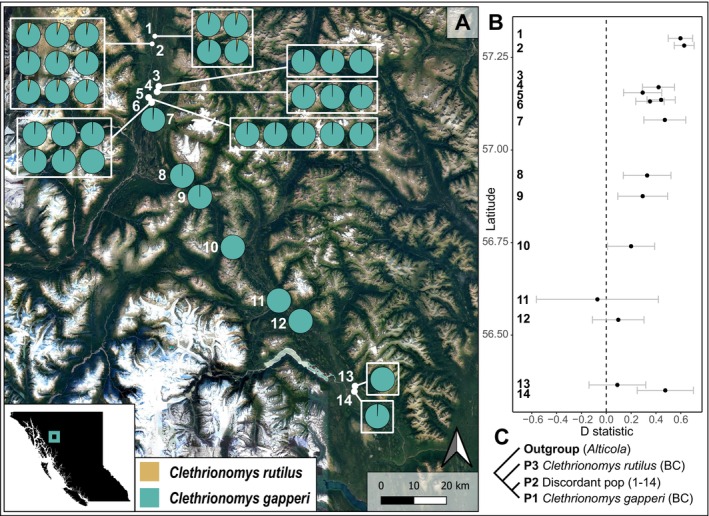
(A) Nuclear ancestry proportions for individuals in the British Columbia contact zone. Each pie chart represents nuclear species ancestry as inferred by *STRUCTURE* (*K* = 2) for each individual with mitonuclear discordance (i.e., each individual has a *C. rutilus* mitogenome paired with majority *C. gapperi* nuclear ancestry). Individuals collected within 150 m of each other are grouped into populations, labeled 1–14. (B) The *D* statistic was calculated for each labeled population using ABBA‐BABA tests, such that a positive value indicates nuclear introgression from *C. rutilus* into that population. The black dots show estimated values of *D* and gray bars indicate 95% confidence intervals. (C) The topology used to perform the ABBA‐BABA tests.

Admixture proportions inferred with *STRUCTURE* (*K* = 2) sorted *C. rutilus* and *C. gapperi* into two distinct genetic clusters (Figure 2A). Only one individual had < 95% assignment to one of the two species clusters, an individual from NWT (FN414) with 76.2% *C. gapperi* ancestry and 23.8% *C. rutilus* ancestry. Triangle plots (Figure 2B–D) validated that individual as a recent hybrid, most likely a first‐generation backcross (Wiens et al. 2025). Virtually all other individuals appeared as parentals on the triangle plots, except for a few *C. gapperi* from BC which had slightly elevated interclass heterozygosity. Interestingly, those individuals all had *C. rutilus* mitochondrial ancestry and slight proportions of *C. rutilus* nuclear ancestry based on *STRUCTURE* results (Figure 2). To investigate further, we calculated *D* to explicitly test whether discordant *C. gapperi* in BC contained small proportions of *C. rutilus* nuclear ancestry. *D* was positive and significant (*p* < 0.05) for populations 1–10 and 14, providing evidence for nuclear introgression (Figure 3B). In general, *D* was highest for the northernmost populations and descended across populations to the south, a pattern that matches observed ancestry proportions (Figure 3A). We also detected evidence of nuclear introgression along the Stikine River in SEAK (*D* = 0.28, *p* = 2.56 × 10^−7^), despite limited support for nuclear admixture in SEAK from *STRUCTURE* and triangle plots (Figure 2).

### 3.3 Intraspecific Genetic Connectivity

**FIGURE 4:**
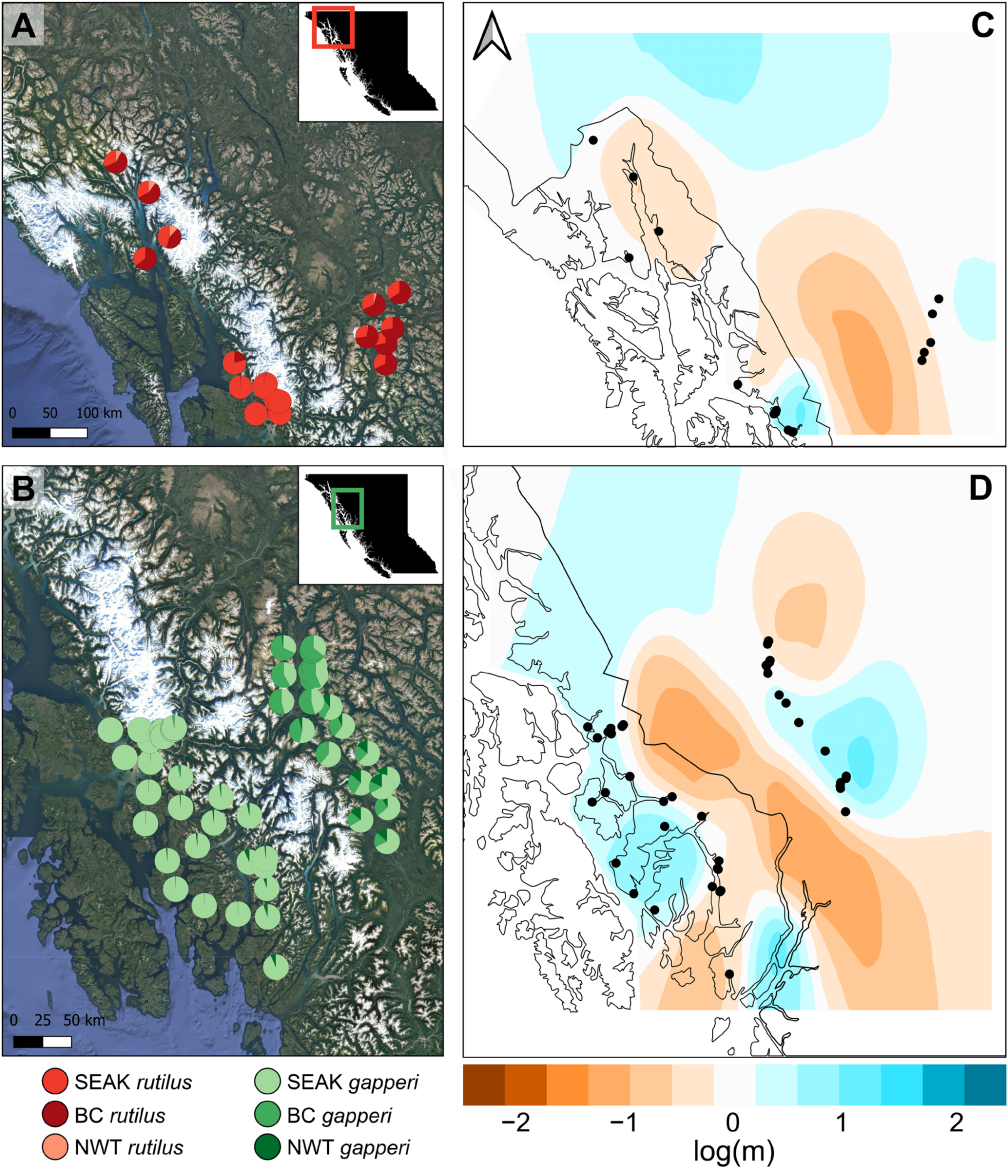
Intraspecific ancestry proportions for (A) *C. rutilus* and (B) *C. gapperi* inferred with *STRUCTURE* (*K* = 3 for each species separately). Only individuals from Southeast Alaska and British Columbia are shown. Each pie chart represents the nuclear ancestry proportions for a single individual. Effective migration surfaces were also built separately for (C) *C. rutilus* and (D) *C. gapperi* , using only individuals from Southeast Alaska and British Columbia. Darker shades of blue indicate regions of higher than expected effective migration (*m*) and darker shades of red indicate regions of lower than expected effective migration.

We detected geographic structuring within both species by running *STRUCTURE* using nuclear SNPs separately for each. Within *C. rutilus* , the NWT and SEAK populations were largely assigned to separate, distinct clusters, while BC and some SEAK individuals displayed mixed intraspecific ancestry (Figure 2A). Mapping those intraspecific ancestry proportions in geographic space revealed that the SEAK *C. rutilus* individuals that cluster with BC occur only in northern SEAK, near the Lynn Canal (Figure 4A). Within *C. gapperi* , mixed intraspecific ancestry proportions across NWT and BC are suggestive of continuous genetic variation across those populations (Figure 2A). Conversely, individuals from SEAK were largely assigned to a single genetic cluster, but a few *C. gapperi* individuals from SEAK and BC shared small proportions of ancestry with the NWT population. Surprisingly, those individuals mapped to the southern parts of the sampled transects in SEAK and BC (Figure 4B). Effective migration maps (Figure 4C,D) corroborate the geographic breaks in intraspecific genetic connectivity inferred from *STRUCTURE*. For both species, the Coast Mountains, which separate coastal SEAK from interior BC, corresponded to lower migration rates than expected given the geographic distance between individuals on either side of the mountains. Within SEAK *C. gapperi* , there is no evidence for breaks in genetic connectivity across islands.

## 4 Discussion

### 4.1 Biogeography

We add to a growing body of literature on mitonuclear discordance, which has been documented across a wide array of taxa (Toews and Brelsford 2012). The case of mitonuclear discordance in red‐backed voles is particularly striking, in that it has arisen in two very different geographical contexts, and provides insight into the biogeographic history of these species since the LGM. Previous work identified different refugial origins for these species: *C. rutilus* in Beringia and *C. gapperi* south of the continental ice sheets in North America (Kohli et al. 2015; Runck and Cook 2005). Both species have colonized previously glaciated land since the LGM, leading to the formation of a broad contact zone across high‐latitude North America. Based on regional patterns of deglaciation, it is likely that contact began at different times across the continent: first in Southeast Alaska, followed by British Columbia, and last in Northwest Territories (see Figures S2–S4 for relevant ice sheet reconstructions and place names).

#### 4.1.1 Red‐Backed Vole Colonization Pathways Into Southeast Alaska

Parts of Southeast Alaska are hypothesized to have remained ice‐free during the LGM, with biogeographic patterns and geological reconstructions pointing to the outer islands of the Alexander Archipelago as the likely location of glacial refugia (Androski et al. 2024; Carrara et al. 2007). *Clethrionomys rutilus* is not present on any islands in Southeast Alaska (except Douglas Island, which is connected by road to the city of Juneau and otherwise separated from the mainland by the narrow and shallow Gastineau Channel), but does occur along the coastal mainland as far south as LeConte Bay (Macdonald and Cook 2007). *Clethrionomys gapperi* occurs on the coastal mainland of Southeast Alaska south of LeConte Bay, as well as on Etolin, Wrangell, and Revillagigedo islands, described as part of the “Middle and Southern Inner Islands” of the Alexander Archipelago (Cook et al. 2006; Macdonald and Cook 2007). Each of those islands is represented by at least one specimen in our RADseq data. For both species, we recover only minimal phylogenetic divergence between British Columbia and Southeast Alaska (Figure 2), supporting previous work that suggests recent colonization of Southeast Alaska by red‐backed voles (Runck et al. 2009).

For individuals with *C. gapperi* nuclear genomes in Southeast Alaska, we find no evidence for population structuring or reduced gene flow across islands (Figure 4). Instead, those analyses highlight the Coast Mountains as a considerable barrier to dispersal between interior British Columbia and coastal Southeast Alaska. Taking into account recent glacial reconstructions (Dalton et al. 2020), we suggest that *C. gapperi* followed a coastal route from the south to colonize Southeast Alaska quickly after that corridor was deglaciated around 14,200 BP (Dalton et al. 2020). Given the poor dispersal ability of red‐backed voles across large bodies of water (Dewsbury et al. 1982), this suggests that the southern interior islands of the Alexander Archipelago may have been connected to the mainland at this point in time, or at least that the straits isolating the islands were narrower than present, allowing *C. gapperi* to colonize Etolin, Wrangell, and Revillagigedo islands. This interpretation is supported by the proximity of those islands to the coastal mainland and the expectation of lower sea levels at a time when much of the high arctic was still glaciated (Shugar et al. 2014). Reconstructions of the postglacial vegetation history on Mitkof Island document a pine‐alder forest established as early as 12,900 BP, supporting the notion that the region was hospitable to *C. gapperi* quickly following glacial recession (Ager et al. 2010). The absence of *C. gapperi* from islands to the north, as well as Prince of Wales Island, despite available habitat as early as ~14,000 BP (Ager 2019), suggests that those islands were not accessible to species colonizing Southeast Alaska via the southern coastal route. Interestingly, we find no evidence for mitochondrial introgression from *C. rutilus* on Etolin, Wrangell, or Revillagigedo islands, despite the existence of *C. rutilus* mitochondria in *C. gapperi* at the same latitudes on the coastal mainland. That pattern suggests the southern islands of the Alexander Archipelago were isolated by rising sea levels before colonization routes opened in the north. Southeast Alaska *C. gapperi* are highly disconnected from populations east of the Coast Mountains in British Columbia, suggesting a later wave of northward expansion by more southern populations of *C. gapperi* as continental North America was slowly deglaciated after Southeast Alaska (Dalton et al. 2020).

The distinct *C. rutilus* mitochondrial lineage that only occurs with coastal mainland *C. gapperi* nuclear genomes raises questions about when and where contact between these species occurred in Southeast Alaska. Previous work also found evidence for this divergent *C. rutilus* mitochondrial haplotype, and interpreted this pattern as evidence for multiple waves of *C. rutilus* colonization into Southeast Alaska (Runck et al. 2009). Given the observed population structure in our nuclear data, we suggest at least one wave of *C. rutilus* colonization out of Beringia and along the Southeast Alaskan coastal mainland once the northern corridor became ice‐free ~12,800 BP (Dalton et al. 2020). The absence of *C. rutilus* from the Alexander Archipelago suggests that the islands were not easily accessible from the mainland at this point (Androski et al. 2024; Mann and Gaglioti 2024). Given the genetic similarity between *C. rutilus* in the Lynn Canal region of Southeast Alaska and those in British Columbia, a second wave could have followed that led to the colonization of both regions, but which failed to extend farther south along the Southeast Alaskan coastline. However, the observed genetic relationships could also be explained by one wave of expansion and continued genetic connectivity between the Lynn Canal region and interior North America. Our sampling gap between the Lynn Canal region and the rest of our *C. rutilus* SEAK samples, however, makes it difficult to reach a strong conclusion about intraspecific *C. rutilus* relationships along the coastal mainland. Nevertheless, it is difficult to envision a scenario whereby the divergent introgressed *C. rutilus* mitochondria can be explained solely by multiple waves of colonization. *Clethrionomys rutilus* populations on the north side of the LeConte Bay do not share the divergent *C. rutilus* mitochondria of *C. gapperi* populations on the south side of the LeConte Bay, despite being separated by < 5 km. If the divergent introgressed *C. rutilus* mitochondria represent an initial wave of colonization, then that wave has been entirely replaced by another wave of *C. rutilus* from the north and *C. gapperi* from the south.

#### 4.1.2 Moving Contact Zone in British Columbia

Contact at the British Columbia transect must have occurred within the last 11,800 years, when the region became completely ice‐free (Dalton et al. 2020). Yet despite deglaciation, postglacial vegetation reconstructions suggest the advancement of boreal forest slowed as it reached northwestern British Columbia (Strong and Hills 2013). Various reconstructions suggest the region was dominated by a shrub and herb assemblage from 9000 BP until ~8000 BP (Spooner et al. 1997, 2002), that spruce arrived around 8200 BP (Mazzucchi 2001), and that pine did not reach the region until 4000–5000 BP (Mazzucchi 2001; Spooner et al. 1997). Further, while various tree species may have been present by 8000 BP, they were likely sparse and did not reach modern densities until 5000 BP (Macdonald 1987). While the current location of the red‐backed vole contact zone in British Columbia was deglaciated by 11,800 BP, suitable habitat, especially for the primarily forest‐associated *C. gapperi* , may not have arrived until much later (Merritt 1981).

Given minimal divergence of introgressed *C. rutilus* mitochondrial haplotypes from non‐introgressed *C. rutilus* mitochondrial haplotypes, our data suggest that contact in BC began relatively recently, with expanding populations of each species likely tracking the postglacial advancement of the boreal forest. We propose that since initial contact, *C. gapperi* has continued to expand north, capturing the *C. rutilus* mitogenome as it does so. While we do not have direct evidence for northward expansion of *C. gapperi* populations, a few lines of evidence support this hypothesis. First, northward expansion is in line with expectations for species in temperate environments in response to climate change since the LGM (Hewitt 2000). Second, the highest proportions of nuclear admixture are found in the northernmost *C. gapperi* (discordant pop 1 in Figure 3), and *C. rutilus* ancestry dissipates in discordant populations to the south. Third, the six voles sampled just 16 km north of discordant pop 1 have *C. rutilus* nuclear and mitochondrial genomes, suggesting that the species are actively hybridizing somewhere within that 16 km region. Further, out of 142 individuals with nuclear and mitochondrial data, we do not detect a single instance of mitochondrial introgression from *C. gapperi* into *C. rutilus* . Together, these patterns support a moving contact zone, wherein *C. gapperi* has captured the *C. rutilus* mitogenome as the center of the contact zone continues to expand north. This process has generated a wake of mitonuclear discordance, which has also been observed in other cases of moving contact zone examples (Chatfield et al. 2010; Krosby and Rohwer 2008; Leaché et al. 2017, 2025; Sequeira et al. 2020).

#### 4.1.3 Parapatry in the Northwest Territories

Red‐backed voles seem to exist in parapatry at our sampled contact zone in the Northwest Territories, with the Kakisa River dividing *C. rutilus* to the north from *C. gapperi* to the south. Evidence for near‐complete parapatry in this region has been found before. Through extensive surveys along the Kakisa River from 1961 to 1976, McPhee (1977) found that only 10 of 407 red‐backed voles trapped on the south bank were *C. rutilus* , and only 8 of 277 trapped on the north bank were *C. gapperi* . Dispersal across the Kakisa River could be occurring during winter months, when the river freezes over (McPhee 1977). Alternatively, the Kakisa River bridge, built in the 1960s during construction of Highway 1 and located at our sampling site, could also facilitate cross‐river dispersal. Interestingly, despite known river crossings since the 1960s, we find both species remain on their respective banks of the river with almost no evidence of admixture. Of the 20 red‐backed voles we sequenced from the Kakisa River region, there was no evidence of mitonuclear discordance and only one individual showed evidence for nuclear admixture, a first‐generation backcross to *C. gapperi* sampled on the south bank. So while these species are capable of producing at least one generation of viable offspring, and despite contact since at least the 1960s, these species have remained genetically isolated across the Kakisa River. One explanation for this pattern is simply that the Kakisa River provides enough of a barrier to dispersal so as to prevent admixture, despite occasional river crossings. If only 2%–3% of red‐backed voles on either side of the river are of the other species (McPhee 1977), it may be difficult for them to find a mate. Even if interspecific breeding did occur, introduced genetic material would be at such a low frequency that it could quickly be purged through drift alone (Kimura and Ohta 1969). If there is any form of selection against interspecific mating, such as through species recognition, decreased hybrid fitness, or asymmetric genetic incompatibility, introgression of nuclear or mitochondrial ancestry would be even more difficult (Bonnet et al. 2017). Alternatively, the lack of significant introgression across the Kakisa River could be indicative of this being a more recent contact zone than those in British Columbia and Southeast Alaska. Deglaciation and postglacial vegetation reconstructions provide some support for that scenario (Dalton et al. 2020; Moser and MacDonald 1990; Seppä et al. 2003). Such a case would offer an interesting case study about the repeatability of evolution. Given enough time, will unidirectional introgression of *C. rutilus* mitochondria into the *C. gapperi* nuclear background arise as it did in British Columbia and Southeast Alaska?

### 4.2 Mitonuclear Discordance

Mitonuclear discordance is observed extensively across the tree of life, leading to various proposals for the mechanisms that drive this phenomenon (Sloan et al. 2017). In general, there are two evolutionary processes capable of generating mitonuclear discordance: ILS and hybridization (Toews and Brelsford 2012). We are able to rule out ILS as the driver of mitonuclear discordance in red‐backed voles, because discordance is localized to geographic regions where the species are in proximity (Funk and Omland 2003; Toews and Brelsford 2012), and because there is also evidence of nuclear introgression. Instead, such patterns are indicative of hybridization as the driver of mitonuclear discordance. Population genetic and ecological theory provide insight into the mechanisms through which mitonuclear discordance can arise through hybridization, and we explore possible explanations in the case of red‐backed voles through the lens of biogeography.

#### 4.2.1 A Divergent Discordant Mitochondrial Clade in Southeast Alaska

Given that Southeast Alaska was deglaciated and forested before regions to the east of the Coast Mountains in British Columbia, we expect that hybridization occurred in Southeast Alaska first, perhaps thousands of years before contact occurred in British Columbia and the Northwest Territories. The result of hybridization in Southeast Alaska is unidirectional mitochondrial introgression of a divergent *C. rutilus* mitochondrion, which appears to have swept to fixation in the Stikine River region. Our mitochondrial phylogeny based on only a few RAD loci from the mitochondrial genome does not provide sufficient resolution to confidently infer the exact relationship of the introgressed *C. rutilus* mitochondria in relation to those that occur in full *C. rutilus* individuals. Previous work using partial *cytochrome b* sequences also detected the divergent introgressed *C. rutilus* mitochondria described here, but was also unable to infer precise phylogenetic mitochondrial relationships (Runck et al. 2009). While it is possible that the divergent introgressed *C. rutilus* mitochondrial lineage reflects multiple waves of colonization into Southeast Alaska, we propose another mechanism through which this pattern may have arisen. Mitochondrial genes must interact smoothly with at least some of the ~1100 N‐mt genes for key organismal processes including oxidative phosphorylation, aerobic respiration, and energy metabolism to function properly (Gaertner et al. 2025; Rath et al. 2021; Weaver et al. 2022). When mitochondria introgress across species boundaries, they are separated from coevolved N‐mt alleles, and mitochondrial function may be impaired (Sloan et al. 2017). Evidence for decreased fitness due to mitonuclear incompatibilities has been found in other species, including mice (Ma et al. 2016), birds (McDiarmid et al. 2024), swordfish (Moran et al. 2024), and copepods (Lima et al. 2019; Pereira et al. 2021). Given the propensity of mitochondria to evolve rapidly (Allio et al. 2017; Harrison 1989), it is possible that the divergent introgressed *C. rutilus* mitochondria can be explained by rapid molecular mitochondrial adaptation to *C. gapperi* N‐mt alleles. Yet, if the introgressed *C. rutilus* mitochondria evolved to adapt to the new nuclear background, then that raises questions about how a lower‐fitness ancestral mitochondria became established in the first place. Larger sample sizes and complete mitogenome sequences will be needed to fully unravel the history of introgression in this region.

#### 4.2.2 Mechanisms for Unidirectional Mitochondrial Introgression in British Columbia

In British Columbia, we found evidence for unidirectional mitonuclear introgression from *C. rutilus* into *C. gapperi* spanning ~120 km across a north–south gradient. Various genetic, demographic, and selective forces can be invoked to explain that pattern. A genetic explanation for unidirectional mitochondrial introgression relies on the possibility of asymmetric reproductive incompatibilities from reciprocal crosses (Sloan et al. 2017). Darwin's corollary to Haldane's rule states that the offspring of reciprocal crosses of the same two species are often not equally fit (Turelli and Moyle 2007). In the case of red‐backed voles, if the offspring of male *C. gapperi* and female *C. rutilus* crosses are viable but the offspring of the reciprocal cross are not, then only *C. rutilus* mitochondria could introgress across species boundaries. While the two species have the same diploid number of chromosomes (*N* = 56), they have different forms of the Y chromosome, which could give rise to asymmetrical reproductive incompatibilities (Rausch and Rausch 1975). Laboratory crosses would be needed to verify the existence of asymmetric incompatibilities, although anecdotal evidence from breeding trials in the 1970s suggests ♂ *C. rutilus* × ♀ *C. gapperi* crosses are capable of yielding viable offspring (McPhee 1977). If true, this suggests that other mechanisms are driving unidirectional mitochondrial introgression in this system.

A common demographic explanation for unidirectional mitochondrial introgression is male‐biased dispersal of an invading species, which results in the spread of nuclear ancestry but not mitochondria (Melo‐Ferreira et al. 2009; Sloan et al. 2017; Toews and Brelsford 2012). There is solid evidence for male‐biased dispersal in a few species of voles (Gauffre et al. 2009; Ishibashi and Saitoh 2008), and observational evidence in *C. rutilus* and *C. gapperi* (McPhee 1977), suggesting that male‐biased dispersal may have contributed to mitonuclear discordance in British Columbia. The biogeographic histories of these species, as inferred here and elsewhere (Kohli et al. 2015; Runck and Cook 2005), lend additional weight to this argument. *Clethrionomys gapperi* is inferred to have speciated in boreal forests south of the continental ice sheets during Pleistocene glacial cycles (Kohli et al. 2014; Runck and Cook 2005). Northward expansion from southern forests has led to contact with *C. rutilus* , the latter of which expanded southeast out of Beringia and is largely associated with tundra and taiga‐like environments (Kohli et al. 2015). The two species currently contact in the boreal forest of northern British Columbia, and if the main factor regulating *C. gapperi* expansion is suitable habitat, regardless of the presence of *C. rutilus* , then continued expansion into the range of *C. rutilus* is likely. Combined with male‐biased dispersal, a moving contact zone facilitated by continuous northward expansion by *C. gapperi* might be able to explain unidirectional mitochondrial introgression in British Columbia. Yet, simulations suggest that sex‐biased demographic processes alone are not capable of producing unidirectional mitochondrial introgression (Bonnet et al. 2017). Further, without direct evidence for male‐biased dispersal in red‐backed voles, it is worth considering alternative mechanisms for unidirectional mitochondrial introgression, which cannot be explained by a moving contact zone alone.

The completeness of mitochondrial introgression into *C. gapperi* populations to the immediate south of the current contact zone suggests mitonuclear discordance in British Columbia might not be governed solely by nonadaptive processes (e.g., male‐biased dispersal, Darwin's corollary). While long thought to be selectively neutral (Moritz et al. 1987), mitochondrial genomes code for proteins that play key roles in energy production and metabolism (Calvo et al. 2016; Weaver et al. 2022), and multiple studies have suggested mitochondria may face strong selective pressure (Ballard and Whitlock 2004; Hill et al. 2019; Lajbner et al. 2018; Rand 2001). Species that evolved at high latitudes during glacial cycles, like *C. rutilus* , would have faced strong selective pressure for elevated endogenous heat production in extremely cold environments (Wallace 2007). There is evidence for mitochondrial thermal adaptation in other species at high latitudes, including in icefishes (Minhas et al. 2023; O'Brien and Mueller 2010) and humans (Wallace 2007). Further, some support for adaptive mitochondrial introgression has been found for Brook charr (Doiron et al. 2002) and hares (Melo‐Ferreira et al. 2014) in response to thermal regimes. If the *C. rutilus* mitogenome is better adapted for cold environments, it may also convey a selective advantage for *C. gapperi* as the species expands north, which could explain the observed pattern of unidirectional mitochondrial introgression. Adaptive mitochondrial introgression provides a mechanism through which the observed patterns of mitochondrial and nuclear ancestry in British Columbia can be readily explained, but more work is needed to test this hypothesis directly.

### 4.3 Future Directions

Various models rooted in population genetic and ecological theory have been proposed to describe patterns of mitonuclear discordance, as seen here and in other systems (Bonnet et al. 2017; Sloan et al. 2017; Toews and Brelsford 2012). Given the limitations of our dataset (i.e., reduced representation of the nuclear and mitochondrial genomes) we are unable to directly test those models, but the increasing tractability of population genetics with whole genome resequencing data could facilitate such research avenues. In theory, mitochondrial introgression should be highly disadvantageous because co‐evolved mitonuclear gene networks are broken up (Hill et al. 2019; Weaver et al. 2022). Therefore, the persistence of mitonuclear discordance in this and other systems raises a series of questions: How do populations adapt to mitonuclear discordance? Can heterospecific mitochondria be more fit than conspecific mitochondria in certain environments? Must co‐adapted nuclear alleles be retained when mitochondria introgress across species boundaries? Can introgressed mitochondria mitigate selection against disrupted gene networks through rapid adaptive evolution? Population genomics represents a promising approach to unraveling the paradox of mitonuclear discordance.

## Author Contributions

**Ben J. Wiens:** conceptualization (equal), formal analysis (lead), writing – original draft (lead). **Jocelyn P. Colella:** conceptualization (equal), writing – review and editing (lead).

## Funding

The Beringian Coevolution Project (NSF#0196095 and NSF#0415668) contributed to specimen collection. A Doctoral Student Research Fund award provided by the University of Kansas contributed to the generation of RADseq data. This work was supported in part by the James D. and Susan E. Smith Endowment for Mammalogy at KU.

## Conflicts of Interest

The authors declare no conflicts of interest.

## Supporting information


**Figure S1:** ece372603‐sup‐0001‐FigureS1.pdf.

**Data S1:** ece372603‐sup‐0002‐DataS1.xlsx.

**Data S2:** ece372603‐sup‐0003‐DataS2.docx.

## Data Availability

Data accessibility: All specimens used in this study are reported in Data S1 and are archived in one of four natural history collections: University of Kansas Biodiversity Institute (KU), Museum of Southwestern Biology (MSB), University of Alaska Museum of the North (UAM), or University of Wisconsin Zoological Museum (UWZM). All specimen metadata is publicly available online through each museum's digital database. All RADseq raw reads are available on the NCBI Sequence Read Archive (SRA) under BioProject PRJNA1355180. All code used for data analysis is available at https://github.com/omys‐omics/RBV_RADseq. Benefit sharing: Benefits from this research accrue from the sharing of our data and results on public databases and the public availability of biological specimens archived in research collections for continued research.
