# Supplementary figures and images for: Unidirectional Mitochondrial Introgression Despite Limited Nuclear Admixture in North American Red‐Backed Voles, Clethrionomys rutilus and C. gapperi
Source: Ecol Evol. 2025 Nov 30;15(12):e72603. doi: 10.1002/ece3.72603 (PMC12665433; doi:10.1002/ece3.72603)

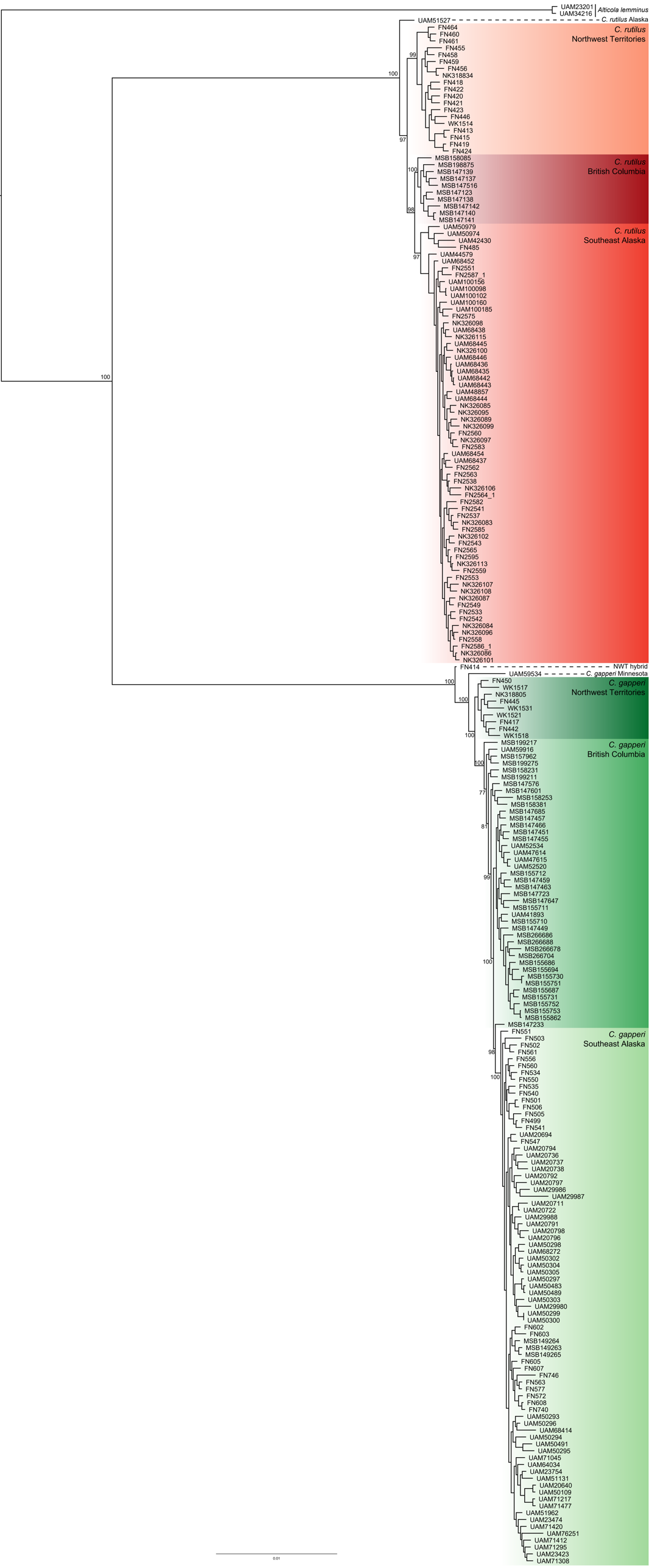

Supplement: Supplementary file 1 — Figure S1: ece372603‐sup‐0001‐FigureS1.pdf. [file ECE3-15-e72603-s002.pdf]
