# Supplementary material for: Unidirectional Mitochondrial Introgression Despite Limited Nuclear Admixture in North American Red‐Backed Voles, Clethrionomys rutilus and C. gapperi
Source: Ecol Evol. 2025 Nov 30;15(12):e72603. doi: 10.1002/ece3.72603 (PMC12665433; doi:10.1002/ece3.72603)
Supplement: Supplementary file 3 — Data S2: ece372603‐sup‐0003‐DataS2.docx. [file ECE3-15-e72603-s003.docx]

**SUPPLEMENTAL INFORMATION FOR:**

**Unidirectional mitochondrial introgression despite limited nuclear admixture in North American red-backed voles, *Clethrionomys* *rutilus* and *C. gapperi***

**Figure S1. (See Supplemental File S1)** Maximum likelihood phylogenetic tree built using all individuals in the nuclear dataset. This tree is identical to the one shown in Figure 2, but with the tips labeled by the museum catalog number of each individual, unabbreviated branch lengths at the base of each species group, and the outgroup (*Alticola lemminus*) retained. Percent bootstrap support values are shown for major nodes.


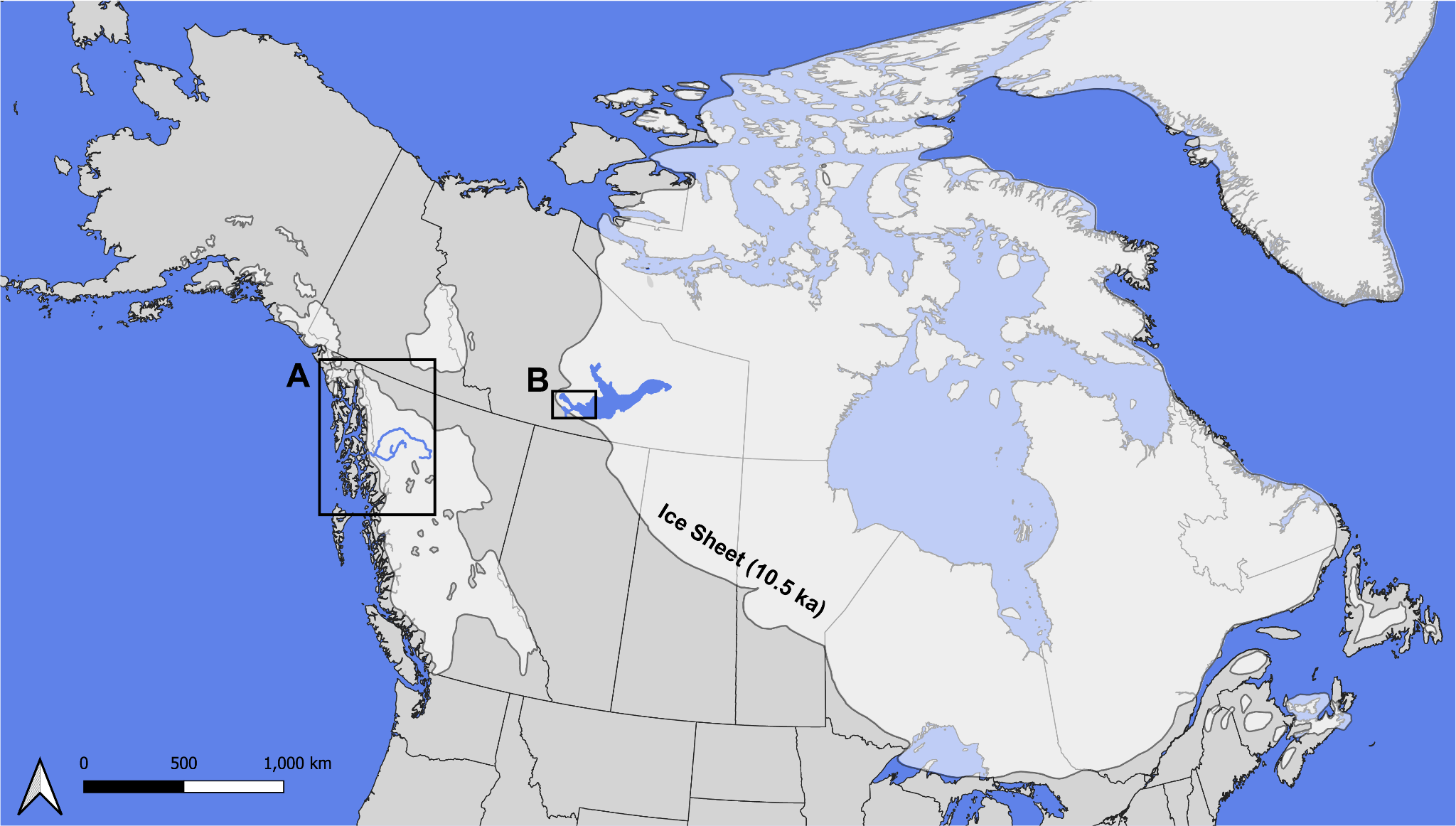


**Figure S2.** Map of North America 10.5 thousand years ago (ka). White shading indicates the estimated extent of continental ice sheets, based on [Dalton et al. (2020)](https://www.zotero.org/google-docs/?DdOr6f). **(A)** The contact zones in Southeast Alaska and British Columbia, shown in more detail in Fig S3. **(B)** The contact zone in Northwest Territories, shown in more detail in Fig S4.


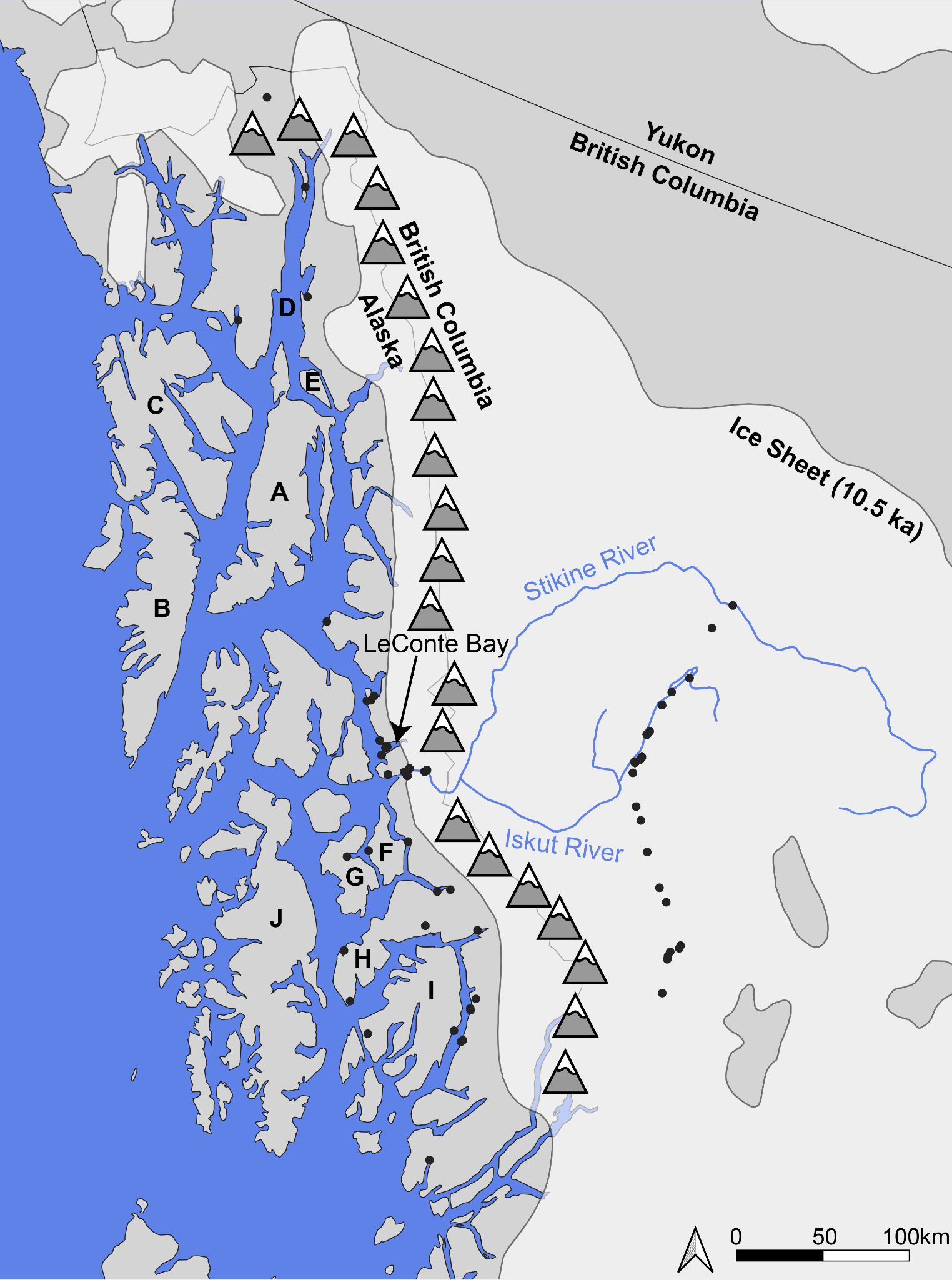


**Figure S3.** Map of the region containing the Southeast Alaska and British Columbia contact zones. White shading indicates the extent of ice sheets 10.5 thousand years ago (ka), based on [Dalton et al. (2020)](https://www.zotero.org/google-docs/?dRRJAT). Black dots indicate red-backed vole specimens used in this study. **(A)** Admiralty Island; **(B)** Baranof Island; **(C)** Chichagof Island; **(D)** Lynn Canal; **(E)** Douglas Island; **(F)** Wrangell Island; **(G)** Etolin Island; **(H)** Cleveland Peninsula; **(I)** Revillagigedo Island; **(J)** Prince of Wales Island. The mountain icons represent the Coast Mountains separating Southeast Alaska from British Columbia.


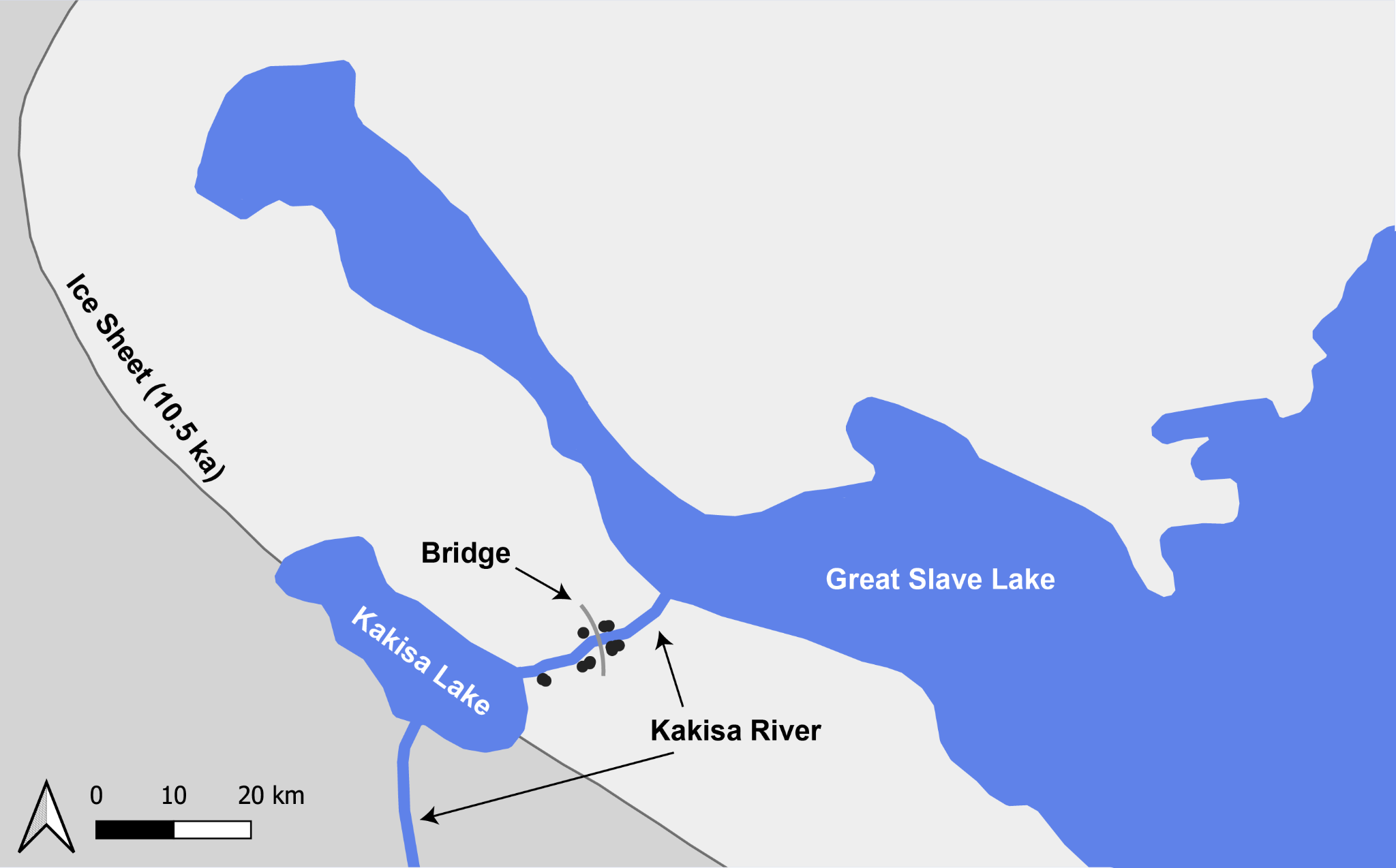


**Figure S4.** Map of the region containing the Northwest Territories contact zone. White shading indicates the extent of the ice sheet 10.5 thousand years ago (ka), based on [Dalton et al. (2020)](https://www.zotero.org/google-docs/?dRRJAT). Black dots indicate red-backed vole specimens used in this study.

*Missing data*

We filtered our nuclear RAD loci heavily for read depth, genotype quality, missingness by SNP, and missingness by individual. These filters removed 14 individuals with high missing data. In the final dataset, 74% of individuals had less than 15% missing data, but there was still a broad distribution of missing data across individuals. We expect that missing data in the retained individuals is largely driven by uneven sequencing effort, which can happen when many individuals are pooled and sequenced together. Indeed, we find a significant association (p=1.615x10^-11^) between raw read count (prior to filtering) and percent missing data across individuals. This suggests that missing data should not heavily influence our analyses, because it is driven by a non-biological factor, sequencing effort. To verify this assumption, we quantified the correlation between missing data and Q1 from the two-species STRUCTURE run, with and without species identity as a co-predictor (Fig S1). Missing data alone explained very little of the observed variance (R^2^=0.003) when used as the only predictor of Q1 (Table S1). When used as a co-predictor with species identity, missing data only explained an additional 4.00x10^-8^ of the observed variance in comparison to species identity alone.

We performed the same exercise for the three q values inferred with each of the intraspecies STRUCTURE runs, with and without geography (e.g. SEAK, BC, NWT) as a co-predictor (Fig S2-S7). Across both intraspecific STRUCTURE runs, missing data explained very little of the observed variance (measured by R^2^) when used as the only predictor (Table S2-S3). When used as a co-predictor with geography, missing data explained an additional 0.5% of the observed variance at most, compared to geography as a predictor alone. Therefore, we expect missing data to have little effect on our results.

**Table S1.** Proportion of observed variance in Q1 explained by missing data in comparison to species identity, as measured by R^2^. Values of Q1 were inferred from the STRUCTURE run with both species. Additional variance explained is the difference between the model that included species identity and missing data in comparison to the model that used only species identity as a predictor.

|  | missing data | species | species + missing data | additional variance explained |
| --- | --- | --- | --- | --- |
| Q1 R^2^ | 0.003067 | 0.999710 | 0.999710 | 4.00x10^-8^ |


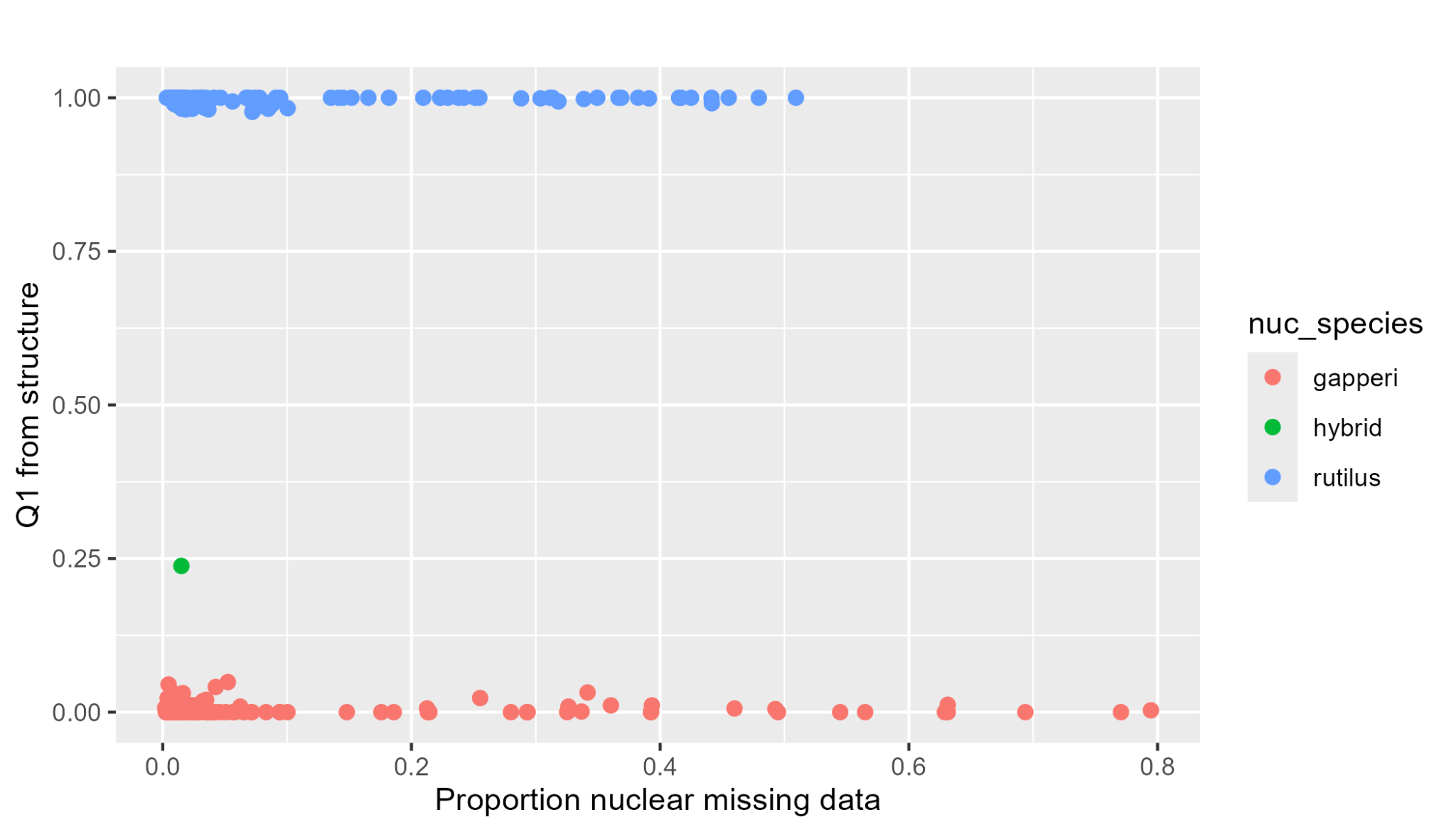


**Figure S5.** The relationship between missing data and Q1 as inferred by STRUCTURE (K=2) for the nuclear SNP dataset including both species (*C. rutilus* and *C. gapperi*).

**Table S2.** Proportion of observed variance in each Q value explained by missing data in comparison to geography, as measured by R^2^. Q values were inferred from the STRUCTURE run with only *C. rutilus*. Additional variance explained is the difference between the model that included geography and missing data in comparison to the model that used only geography as a predictor.

|  | missing data | geography | geography + missing data | Additional variance explained |
| --- | --- | --- | --- | --- |
| Q1 R^2^ | 0.011999 | 0.997520 | 0.997522 | 0.000002 |
| Q2 R^2^ | 0.000069 | 0.781203 | 0.786585 | 0.005382 |
| Q3 R^2^ | 0.009372 | 0.907265 | 0.908963 | 0.001697 |


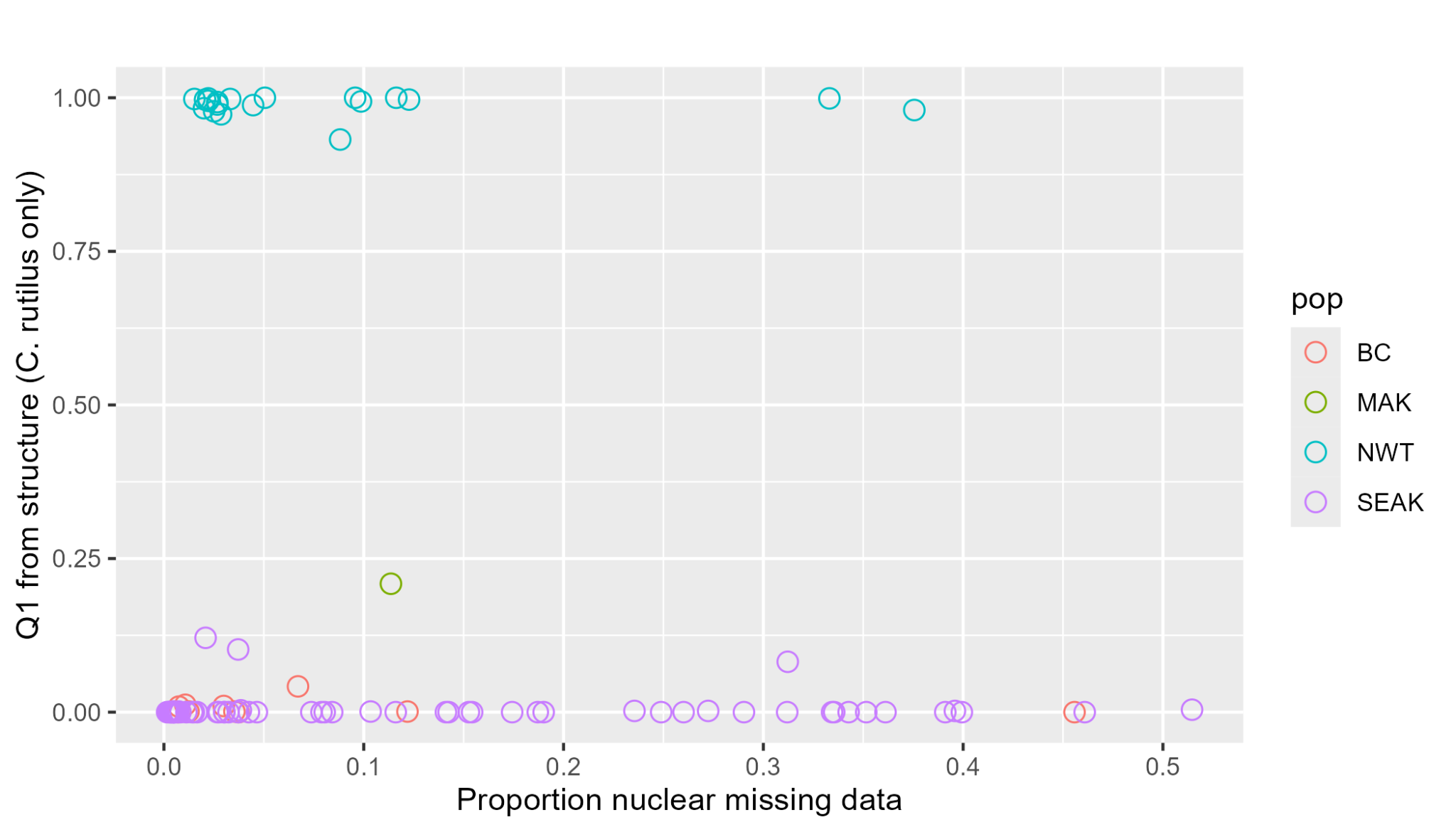


**Figure S6.** The relationship between missing data and Q1 as inferred by STRUCTURE (K=3) for the nuclear SNP dataset for *C. rutilus* only.


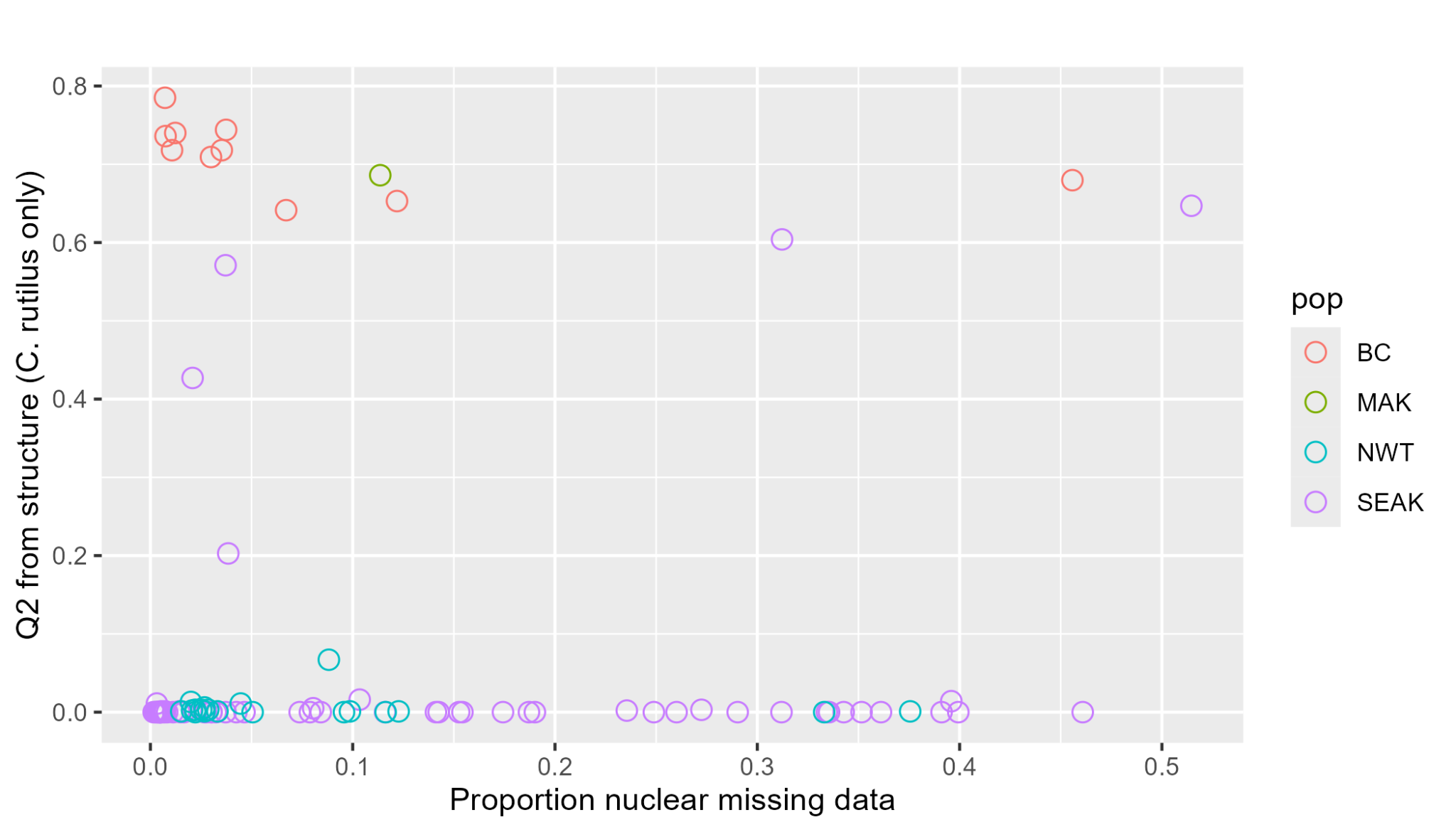


**Figure S7.** The relationship between missing data and Q2 as inferred by STRUCTURE (K=3) for the nuclear SNP dataset for *C. rutilus* only.


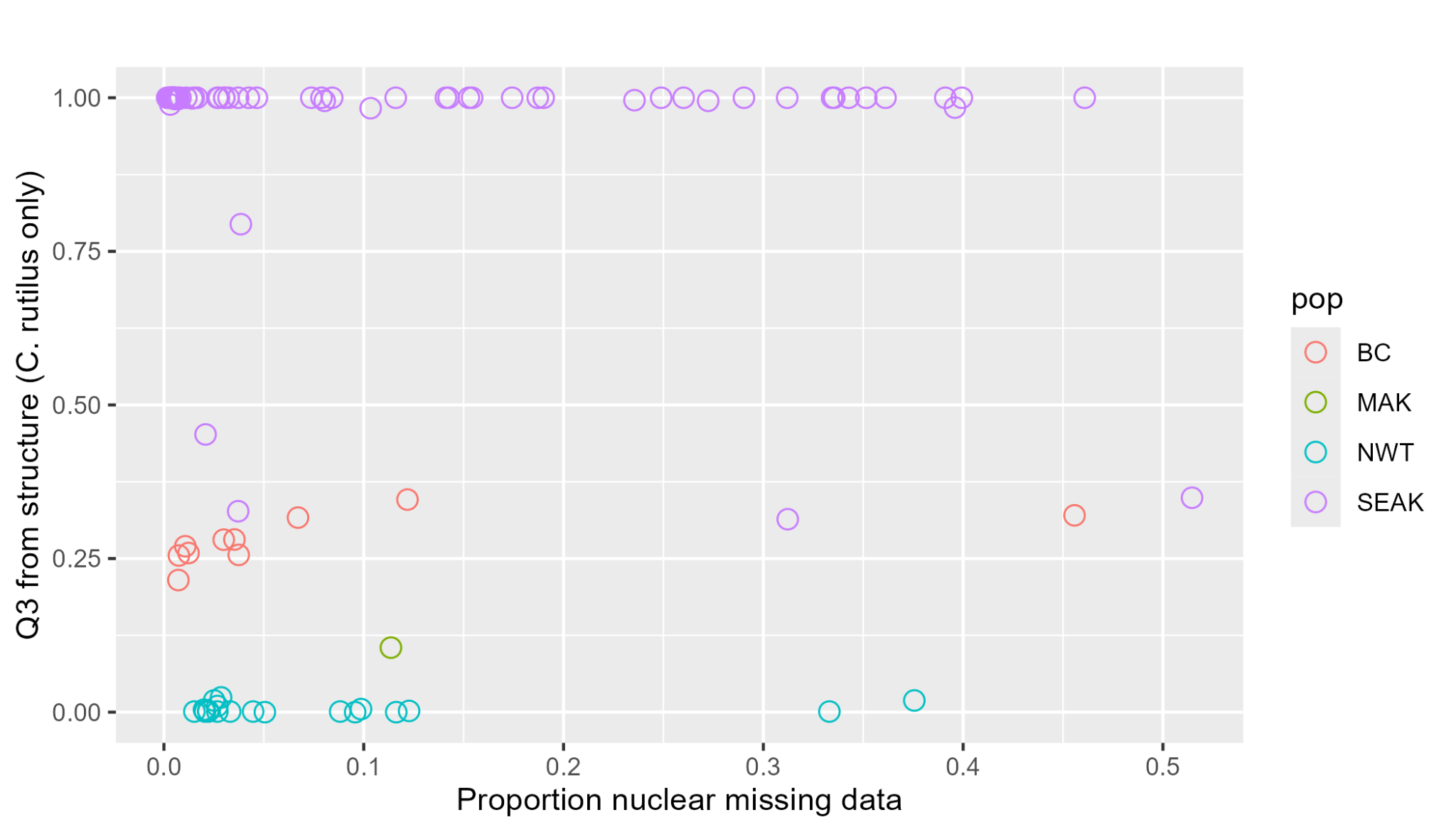


**Figure S8.** The relationship between missing data and Q3 as inferred by STRUCTURE (K=3) for the nuclear SNP dataset for *C. rutilus* only.

**Table S3.** Proportion of observed variance in each Q value explained by missing data in comparison to geography, as measured by R^2^. Q values were inferred from the STRUCTURE run with only *C. gapperi*. Additional variance explained is the difference between the model that included geography and missing data in comparison to the model that used only geography as a predictor.

|  | missing data | geography | geography + missing data | Additional variance explained |
| --- | --- | --- | --- | --- |
| Q1 R^2^ | 0.0345 | 0.92883 | 0.92971 | 0.00088 |
| Q2 R^2^ | 0.02528 | 0.85766 | 0.8582 | 0.00054 |
| Q3 R^2^ | 0.00442 | 0.96151 | 0.96171 | 0.00019 |


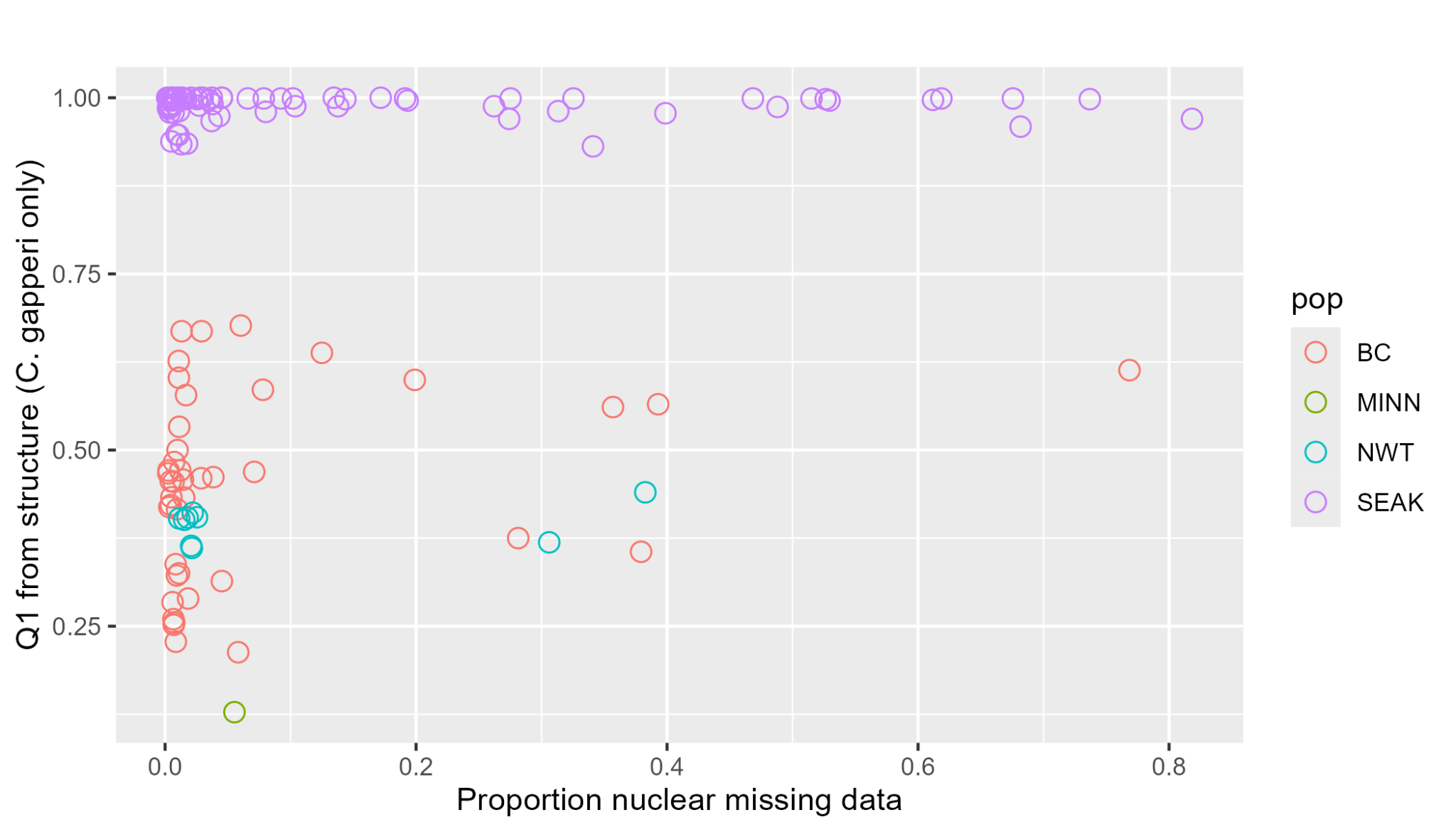


**Figure S9.** The relationship between missing data and Q1 as inferred by STRUCTURE (K=3) for the nuclear SNP dataset for *C. gapperi* only.


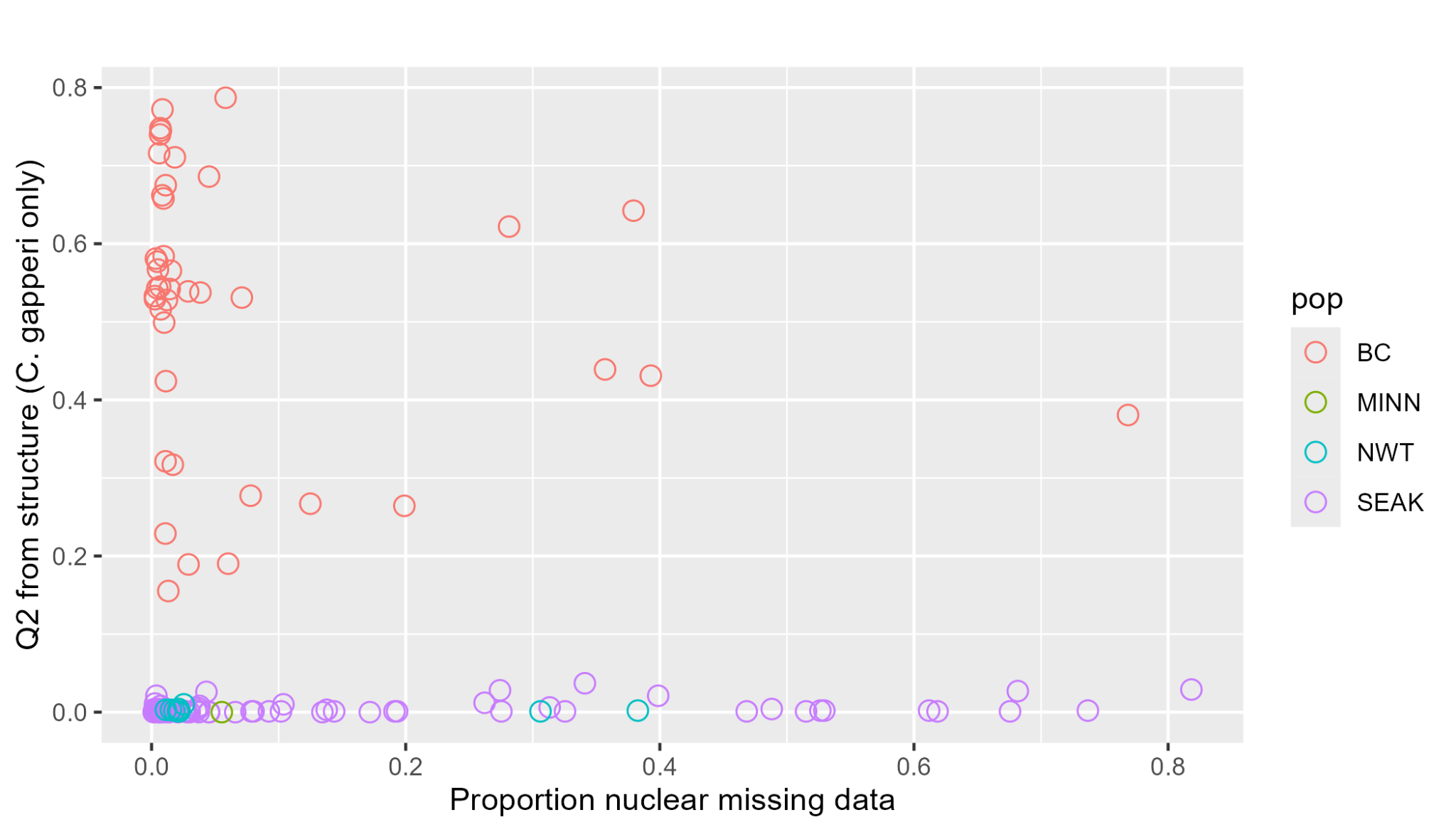


**Figure S10.** The relationship between missing data and Q2 as inferred by STRUCTURE (K=3) for the nuclear SNP dataset for *C. gapperi* only.


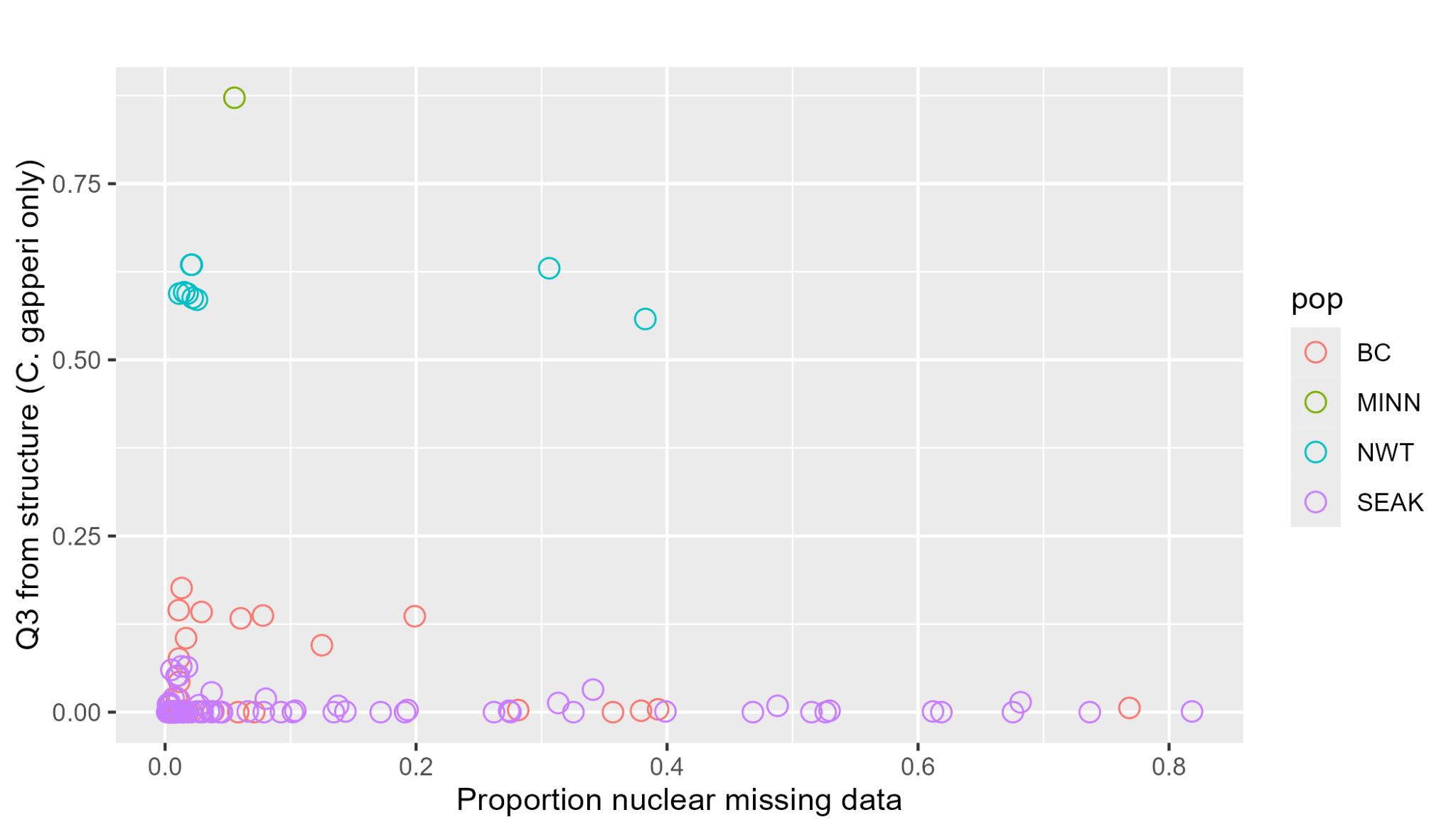


**Figure S11.** The relationship between missing data and Q3 as inferred by STRUCTURE (K=3) for the nuclear SNP dataset for *C. gapperi* only.
